# Prevalence, Clinical Criteria and Sociodemographic Predictors of *Trichomonas vaginalis* Infection in Suspected Egyptian Women, Using Direct Diagnostic Techniques

**Published:** 2015

**Authors:** Atef Hussein HUSSEIN, Mohamed Hussein SALEH, Ibrahim Maged NAGATY, Khaled A GHIETH, Nagat Ahmed EL-AZAB

**Affiliations:** *Department of Parasitology, Behna Faculty of Medicine, Behna University, Benha, Egypt*

**Keywords:** *Trichomonas vaginalis*, Wet mount, Diamond’s culture, Acridine orange

## Abstract

***Background:*** The present study aimed to determine the prevalence and associated risk factors of vaginal trichomoniasis in women referred to gynecologic clinic in Benha University Hospital, Egypt.

***Methods:*** Two hundred female patients enrolled in the study. Vaginal samples were obtained from them and examined for *T. vaginalis *by wet mount, Giemsa stain, Acridine orange (AO) stain and culture on modified Diamond’s medium. For analysis of accuracy of the methods used, the receiver operating characteristic (ROC) curve concept with culture as a gold standard was applied.

***Results:*** Out of 200 patients, *T. vaginalis* was found in 22 (11%) patients by any of the diagnostic methods used. The accuracy of AO staining comes next to Diamond’s culture (AUC 0.909, sensitivity 81.8%, specificity 100%, CI 0.81-1.0) followed by Giemsa staining (AUC 0.835, sensitivity 68.2%, specificity 98.9%, CI 0.72-0.95). The wet mount was the least accurate method (AUC 0.795, sensitivity 59.1%, specificity 100%, CI 0.67-0.92). There was no significant association between potentially supposed risk factors and trichomoniasis except patients complaining of either dysuria and dyspareunia or back pain and abdominal pain.

***Conclusion:*** Trichomoniasis is a common disease in our community. Sociodemographic factors do not seem to affect the prevalence among different Egyptian population. For accurate diagnosis, laboratory investigation is essential. A positive wet smear is diagnostic, but negative samples should be examined by methods that are more sensitive.

## Introduction

*Trichomonas vaginalis* infection is a sexually transmitted disease with important public health consequences. In women, trichomoniasis has a wide spectrum of presentations, from an asymptomatic to an acute or chronic inflammatory disease with a malodorous vaginal discharge (1). Infection is also linked to preterm labour, prenatal morbidity (2). The worldwide incidence of trichomoniasis in 2008 was estimated to be 276.4 million new cases per year (3). Despite this incidence, research and control efforts for *T. vaginalis* infection have traditionally lagged far behind efforts to control other STIs and contributing to its classification as a neglected parasitic infection (4). In Egypt, the reported prevalence rate ranges from 5% to 79.16% (5, 6). Many risk factors linked to infection rate including age, race/ethnicity, education, residence, marital status, number of sex partners, use of condom/IUD, any drug use, history of sexually transmitted diseases and presence of vaginal discharge (7-10). 

Traditionally, diagnosis of *T. vaginalis* infection reached by a wet mount, in which "corkscrew" motility observed (11). However, culture has long been the gold standard for diagnosing *T. vaginalis *infection, with a sensitivity range of 85-95% (12). Other methods used for diagnosis include staining methods (13), latex agglutination (14), enzyme-linked immunosorbent assay (15), immunochromatography and nucleic acid amplification tests (13). In order to develop guidelines for the diagnosis of trichomoniasis, ideal test should have high sensitivity and specificity and be easily available, simple to perform, and inexpensive (16). Almost none of the above-mentioned diagnostic methods do completely fulfill these criteria. 

The aim of the present study was to assess the prevalence of *T. vaginalis* infection and the associated risk factors among women attending the Gynecology and Obstetrics Outpatient Clinic at Benha University hospital, Egypt. In addition, accuracy of direct wet mount and two staining methods (Giemsa and AO) compared using Diamond’s media culture as the gold standard.

## Materials and Methods

***The study participants***

The Study was carried out between August 2013 and February 2014 on 200 non-pregnant female patients attending the Gynecology and Obstetrics Outpatient Clinic at Benha University Hospital, Egypt. The patients were 20 to 50 years old and complaining of variable gynecological complaints including vaginal discharge, itching, lower abdominal pain, backache, dyspareunia or dysuria alone or in combination. The women completed a questionnaire that inquired about age, residence, educational level, marital status, parity and menstrual and contraceptive history. Patients under treatment of vaginitis or cervicitis were excluded. 

The study was approved by the local Ethics Committee and all participant gave consent to do vaginal swabbing.

***Sample collection***

**Fig. 1:**
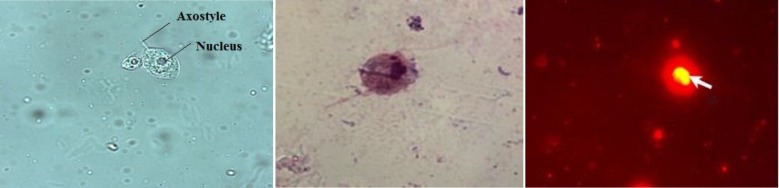
*Trichomonas vaginalis* trophozoite as shown by wet mount smear (left, X630), Giemsa stain (middle, X1000) and Acridine orange (right, trophozoite stained brick red with a yellowish nucleus, X400) (Original)

 Two specimens of vaginal discharge were collected by speculum from the posterior vaginal fornix using sterile vaginal swabs. One ml normal saline was added to first swab and squeezed onto a tube wall to be used, within one hour, for wet mount smear and staining (Fig. 1). The second swab immersed in Diamond's Modified medium culture tube and squeezed for cultivation.

***Wet mount smear***

 One drop of the first swab tube placed onto a microscopic glass slide then covered by a cover slip and examined microscopically within 10-30 min for *T. vaginalis* trophozoite (Fig. 1).

***Giemsa-stained smear*** *(* 17 *)*

 One drop of the vaginal swab-saline suspension smeared on a microscopic glass slide, air- dried, fixed in methanol for 30 minutes, stained with Giemsa dye for 2-3 hours (timing had been modified according to preliminary trials) and then rinsed under gently running water and allowed to dry in a vertical position. The slides examined microscopically at X1000 to detect the violet, pear-shaped trophozoites (Fig. 1).

***AO-stained smear***

One drop of the vaginal swab-saline suspension smeared on a microscopic glass slide, air- dried, heat-fixed and then put in the stain for 20 seconds. The slides were stored in pH 7.2 holding buffer at room temperature in the dark until examined (18). The slides were scanned while wet under fluorescent microscope at X400, using selective beam splitter of TS 510 nm, barrier filter G 247 nm, additional filter of G 249 nm, excitation filter for narrow – band excitation of 255 nm. *T. vaginalis *trophozoites stained brick red with a yellowish-green banana-shaped nucleus (Fig. 1). Yeast and bacteria stained red but significantly smaller and morphologically different, so they were easily distinguishable from trichomonads.

***In vitro cultivation of T. vaginalis***

 The swab specimen inoculated into the pre-warmed media and then incubated at 37 °C for 7 days in anaerobic condition (19). Culture examined daily as wet mount smear.

***Statistical analysis***

 The collected data tabulated and analyzed using SPSS version 16 software (SPSS Inc., Chicago, ILL Company). Data presented as number and percentages. Chi square test (X^2^) or Fisher's exact test used as a test of significance. ROC curve used to determine sensitivity and specificity of direct wet mount, Giemsa stain, and AO stain compared with Diamond’s culture for diagnosis of vaginal trichomoniasis. Two sided *P* <0.05 was considered significant.

## Results

Out of 200 patients investigated, *T. vaginalis* infection was found in 22 (11%) patients by any of the diagnostic methods used. However, only 13 (59.1% of positive cases) were detected by all the four used methods. Diamond’s medium culture detected trichomoniasis in 20 cases (90.9% of positive cases) of which four cases (18.2% of positive cases) were detected only by culture. While 2 cases (9.1% of positive) were detected by Giemsa stain only (Table 1).

**Fig. 2:**
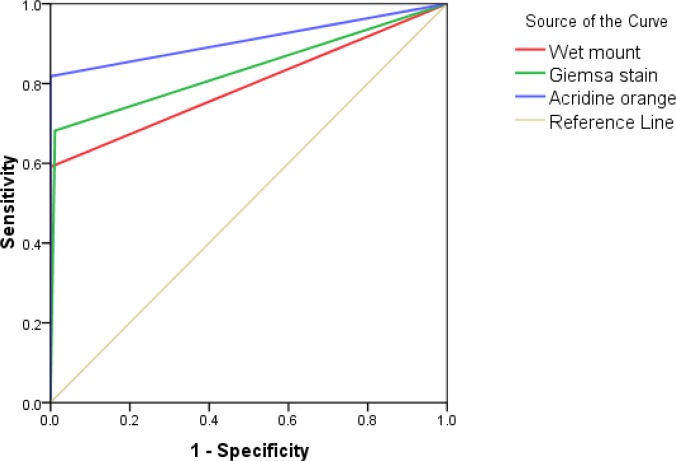
Receiver operating characteristic curve (ROC) analysis of sensitivity and specificity of the methods used in diagnosis of vaginal trichomoniasis

Receiver operating characteristic curve (ROC) and the yielded area under the curve (AUC) analysis of sensitivity and specificity of the methods used in diagnosis of vaginal trichomoniasis is shown in Fig. 2 and Table 1. 

**Table 1:** Accuracy of direct wet mount and staining techniques versus Diamond’s culture, the gold standard test for diagnosis of vaginal trichomoniasis

	**Culture**				**Accuracy measure**			
		**Positive**	**Negative**	**Total**	**Variable**	**%**	**AUC &95%CI**	***P *** **value**
Wet mount	Positive	13	-	13	Sensitivity	59.1		
	Negative	9	178	187	Specificity	100	0.795 &0.67-0.92	<0.001
	Total	22	178	200	Positive predictive value	100		
					Negative predictive value	95.2		
Giemsa stain	Positive	15	2	17	Sensitivity	68.2		
	Negative	7	176	183	Specificity	98.9	0.835 &0.72-0.95	<0.001
	Total	22	178	200	Positive predictive value	88.2		
					Negative predictive value	96.2		
Acridine orange stain	Positive	18	-	18	Sensitivity	81.8	0.909 & 0.81-1.0	<0.001
	Negative	4	178	182	Specificity	100		
	Total	22	178	200	Positive predictive value	100		
					Negative predictive value	97.8		

Given that culture method was used as a gold standard in this study, its sensitivity and specificity were 100% with the area under the curve (AUC) equal 1.0. 

The highest rate of infection among the studied group (23.4%) was significantly (*P*<0.05) found in women having the classical clinical picture of vaginal trichomoniasis (co-mplaining of vaginal discharge, itching, dysuria, dyspareunia, abdominal and back pain) followed by patient complaining of dysuria, dyspareunia and abdominal and back pain (17.2%) and patients complaining of dysuria, dyspareunia (12.5%). Significantly lower prevalence was found among patient complaining of infertility (9%), vaginal discharge (3.1%), vaginal discharge and itching (5.3%). While no single positive case was detected among women represented with itching alone.

Dysuria and dyspareunia are the most frequent symptoms (77.2%) among the positive cases, followed by abdominal and back pain (72.7%), then vaginal discharge (68.2%), then itching 63.3%) and the least frequent symptoms was infertility (4.5%).

Among positive cases, presence of purulent frothy yellowish greenish vaginal discharge is highly significant characteristic (*P* <0.001) of vaginal trichomoniasis. While bloody or watery vaginal discharge is not relevant to trichomoniasis.

Analysis of the socio-demographic profile and clinical criteria of examined suspected women versus detection of *T. vaginalis* infection showed no statistical significant difference except patients complaining of either dysuria and dyspareunia or back pain and abdominal pain that show highly significant statistical difference from other groups (Table 2).

**Table 2:** Socio-demographic profile and Clinical criteria of examined suspected women vs. detection of *Trichomo* *nas vaginalis* infection

	**No. of cases**	**Positive cases (%)**	**Negative cases (%)**	***P*** **-value**
**Age group (yr):**				0.59
20-25	27	2 (7.4)	25 (92.6)	
25-30	49	4 (8.1)	45 (91.9)	
30-35	42	7 (16.6)	35 (83.4)	
35-40	31	5 (16.1)	26 (83.9)	
40-45	32	3 (9.4)	29 (90.6)	
45-50	19	1 (5.2)	18 (94.8)	
**Residence:**				0.88
Urban	88	10 (11.4)	78 (88.6)	
Rural	112	12 (10.7)	100 (89.3)	
**Education level:**				0.59
Higher education	26	2 (7.7)	24 (93.3)	
Primary and 2ry school	102	10 (9.8)	92 (90.2)	
Illiterate	72	10 (13.9)	62 (86.1)	
**Marital status:**				0.38
Married	166	20 (12)	146 (88)	
Unmarried (divorced or widow)	34	2 (5.9)	32 (94.1)	
**Vaginal discharge:**				
Yes	135	15 (11.1)	120 (88.9%)	0.94
No	65	7 (10.8)	58 (89.2%)	
**Dysurea and dysparounia:**				
Yes	84	17 (20.2)	67 (79.8)	<0.001
No	116	5 (4.3%)	111 (95.7)	
**Back and abdominal pain:**				
Yes No	76 124	16 (21.1) 6 (4.8%)	60 (78.9) 118 (95.2)	<0.001
**Itching:**				
Yes	120	14 (11.7)	106 (88.3)	0.71
No	80	8 (10)	72 (90)	
**Infertility:**				
Yes	11	1 (9.1)	10 (90.9)	1.0
No	189	21 (11.1)	168 (88.9)	
**Use of contraceptive:**				
IUD	87	14 (16)	73 (84)	0.13
Hormonal	69	5 (7.2)	64 (92.8)	
No contraception	44	3 (6.8)	41 (93.2)	

## Discussion

The prevalence of vaginal trichomoniasis among women enrolled in this study was 11% (22/200) this corroborates finding by other researchers who reported that among symptomatic women from Cairo and El-Minia cities the prevalence were 10.16% and 12.7%, respectively (20, 21). Higher rate of infection was recorded in Cairo, Mansoura, and Alexandria with prevalence 23%, 79.16% and 79.9%, respectively (6, 22, 23). Globally, prevalence estimates among suspected patients vary between 0.9%-80% (24, 25). The disparity between different studies could be attributed to many factors including variation in selection of the enrolled study population, the sensitivity of the used diagnostic technique or the skill of the investigator. 

The finding recorded in the present study emphasizes the former interpretation. In our study, prevalence rises up to 23.4% (50% of positive cases) in patients having typical vaginal trichomoniasis manifestations (vaginal discharge, itching, dysuria, dyspareunia, abdominal and back pain). On the other hand, the rate of detected positive infection decreased in suspected patient with partial clinical picture. The rate fall down to 3.1% among patients who complain of vaginal discharge only, while no positive cases were detected among patients suffering only from vaginal itching. This result come in agreement with the result of Valadkhani and his colleagues (24) who reported that combination of vaginal discharge, dysuria and itching is the main complaint in 52% of *T. vaginalis* infected patients. While, no positive cases recorded in suspected patients complaining from each separately. In the present study, the most frequent clinical manifestations found among positive cases were dysuria associated with dyspareunia, back pain associated with abdominal pain, vaginal discharge and itching. Moreover, the prevalence of *T. vaginalis *is significantly affected by the characters of the collected discharge. Our data show that, the highest rate of positivity for vaginal trichomoniasis (50%) was found among women having purulent frothy yellowish greenish discharge (41% of total positive cases), while the prevalence was not so high in patient with non-purulent yellowish greenish discharge (12.5%). Bloody and watery vaginal discharges are irrelevant to trichomoniasis. These results agree with the finding of Fernando and his colleagues who reported that in 47% of women with vaginal discharge, it was a characteristic yellowish, frothy and malodorous discharge (26). 

Our data show that, except for patients complaining of either dysuria and dyspareunia or back pain and abdominal pain, there is no association between trichomoniasis prevalence and age, residence, education, marital status, use of contraceptive, presence of vaginal discharge and chronic diseases. This partially agrees with the previous findings (7, 26, 27). The sociodemographic and clinical criteria associated with trichomoniasis are debatable. Many studies reported association between risk of *T. vaginalis* infection and age (28), race/ethnicity (29), marital status (9), education, use of condom/IUD, presence of vaginal discharge (8), any drug use and history of sexually transmitted diseases (10). 

Because the major symptoms of trichomoniasis are nonspecific (24), laboratory techniques are recommended for diagnosis of the infection. Accurate diagnosis of *T. vaginalis* affected by many variables, including patient factors, clinician's experience, specimen sampling, processing and test interpretation as well as the skill set and expertise of those doing microscopic assessments. 

To date, the most common method for *T. vaginalis* diagnosis in women remains microscopic evaluation of vaginal wet preparations due to its low cost and simplicity (30). In the present study, although wet mount is the least accurate test when compared with culture technique (AUC 0.795), it is proved to be highly specific methods. Our results came in accordance with other reports that indicated that wet mount is less sensitive than culture method (20, 31, 32). 

The use of staining methods in *T. vaginalis* infection diagnosis is justifiable when it is not possible to employ the wet mount in proper time. In the present study, the sensitivity of AO was 81.8% compared to the culture. The calculated AUC (0.909) revealed that AO accuracy comes directly next to that of Diamond’s culture. AO staining technique is relatively simple to carry out and shows reasonable sensitivity and specificity (18, 23, 33), but it requires the use of a fluorescent microscope which is not readily available in all settings, particularly in developing countries with limited resources like Egypt. 

Our data shows that, although Giemsa staining method is more sensitive than direct wet mount but still less accurate than AO and culture methods. Radonjic et al. and Ojuromi et al. reached the same conclusion (18, 34). The two positive cases, which detected only with Giemsa method, could be considered false positives as diagnosis depends only on size and shape and not on motility of the parasites as the case in wet mount and culture methods.

The current study shows that culture methods remain the gold standard for diagnosis of *T. vaginalis* infection. Our result comes in compliance with other authors who report and confirm that culture remains the most reliable method in the diagnosis of *T. vaginalis* infection (18). One limitation of culture method is that it does not allow same day treatment. In many developing countries where the cost of return to the health facility can be substantial, patients may not bother to return for their culture results, thus prolongation of the infection, leading to further transmission and the possibility of squeal (16).

## Conclusion

*T. vaginalis* infection is a common disease in our community. In contrary to some other countries, sociodemographic factors do not seem to affect the prevalence among different Egyptian population. Clinical picture is not reliable for accurate diagnosis of *T. vaginalis* infection, thus laboratory investigation is essential. A positive wet smear is diagnostic but negative samples should be examined by more sensitive methods such as culture or AO.
